# Mate value, intrasexual competition and sociosexual desire drive Brazilian women's well-being

**DOI:** 10.1017/ehs.2021.18

**Published:** 2021-03-10

**Authors:** Anthonieta Looman Mafra, Renata Pereira Defelipe, Marco Antonio Correa Varella, John M. Townsend, Jaroslava Varella Valentova

**Affiliations:** 1 Department of Experimental Psychology, University of São Paulo, Avenida Professor Mello de Morais, 1721 Butantã, São Paulo, SP 05508-030, Brazil; 2 Department of Anthropology, Syracuse University, Syracuse, New York, USA

**Keywords:** Depression, happiness, life satisfaction, mating, parenting

## Abstract

Well-being (vs. ill-being) might function as an internal guide for approaching (vs. avoiding) situations, strategies, and achievements that ancestrally led to higher (vs. lower) reproductive success. Indeed, coupled individuals report higher well-being than singles, while depressive individuals report lower mate value and higher sociosexuality. Here we investigate associations between well-being, depression and evolutionary reproduction-related aspects (mate value, intrasexual competition, age, and sociosexuality). Overall, 1,173 predominantly heterosexual Brazilian women (mean = 31.89; standard deviation = 11.10) responded to online instruments measuring self-perceived happiness, life-satisfaction, depression, mate value, intrasexual competition, age, and sociosexuality. Multiple regression models indicated that higher well-being was positively predicted by mate value and negatively by intrasexual competition and sociosexual desire, while the opposite was true for depression. Although intrasexual competition and unrestricted sociosexuality can, under some circumstances, increase individual reproductive success, they are risky and suboptimally effective strategies, thus leading to feelings of ill-being. Contrarily, affective long-term bonds, higher mate-value, and lower intrasexual competition might increase feelings of well-being, because this would lead to a safer route towards ancestral reproductive advantages.

## Graphical abstract

**Figure:**
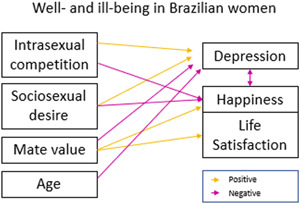

**Social media summary:** Well-being rises with mate value and decreases with sociosexual desire and intrasexual competition in Brazilian women.

## 1. Introduction

Feelings of well-being facilitate individuals’ life goals and prompt them to explore new situations and interact with others (Kenrick & Krems, 2018). In contrast, depressive episodes lead individuals to remain quiet and isolated, preventing individuals from adverse social situations and from confronting higher status individuals (Watson & Andrews, 2002; Workman & Reader, 2014). Such feelings of well- and ill-being might be triggered by external factors, including those concerning the reproductive domain (Troisi, 2008). When asked about the importance of evolutionary-oriented life goals (self-protection, disease avoidance, affiliation, status seeking, mate seeking, mate retention, parental investment) on their well-being, people indicated goals linked to long-term relationships (mate retention and parental investment) as the most important ones, while they ranked mate seeking as the least important (Ko et al., 2019). Indeed, adult individuals, on average, invest more in parenting than mating (Valentova et al., 2020), although, depending on specific conditions, both short- and long-term relationships can bring reproductive advantages (Buss & Schmitt, 2019).

Humans can pursue a mixed mating strategy, shifting between long- and short-term strategies (Gangestad & Simpson, 2000), or they can invest in both mating and parenting at the same time (Valentova et al., 2020). By expanding the number of sexual partners, individuals can increase reproductive fitness by securing immediate resources and a genetic diversity of offspring that receive lower parental investment. Contrarily, by maintaining a mate with parental abilities to share child rearing, committed relationships can increase reproductive success by increasing paternal certainty and the survival rate of fewer offspring who receive higher parental investment (Troisi, 2008).

Short- and long-term relationships are partly influenced by trade-offs between characteristics that define one's value in the mating market and characteristics desired in a partner (Muggleton & Fincher, 2017). Individuals’ mate value and intrasexual competition are two reproductive variables supposed to secure a higher-quality mate, which might enhance one's reproductive success. Although these two variables may improve reproductive success, both work differently in the process of gaining and maintaining a partner. Individuals with higher mate value are mostly targets of desire and competition by potential partners; consequently, they tend to mate with similarly high-mate-value individuals (Kirsner et al., 2003). People with lower mate value compete more than higher-mate-value individuals to acquire a romantic partner with higher mate value (Fisher & Fernández, 2017). However, lower-mate-value individuals are less likely to retain long-term relationships with higher value mates and tend to be less selective (Lee et al., 2008). Thus, intrasexual competition would have a more negative impact on individuals with lower mate value. By correctly assessing their own and their rivals’ mate value, individuals with lower mate value are less prone to engage in a competition with a rival who has a much higher mate value – thus avoiding wasted resources and time (Fisher & Fernández, 2017). Indeed, in men self-rated attractiveness correlates with other-rated attractiveness; however, this does not apply to women (Pereira et al., 2019).

Age is another component that influences attractiveness, particularly in women. Because of their high obligatory initial physiological investment in the offspring and eventual menopause, women have a much shorter window of reproduction than do men (Mafra, 2019). Age is thus a potent limiting factor of female fertility (Mafra, 2017). The nubility hypothesis states that women with higher residual reproductive value would be preferred as reproductive partners (Marlowe, 1998). Indeed, older women are rated as less attractive than men and women of various ages (Foos & Clark, 2011; Maestripieri et al., 2014).

The adoption of specific mating strategies is flexible and depends on environmental demands, personal qualities and individual developmental pathways (Buss & Schmitt, 2019; Gangestad & Simpson, 2000). For example, women, who are on average more sexually restricted than men (Schmitt, 2005, Varella et al., 2014), may become more unrestricted in a society with more gender equality owing to both cultural and biological factors (Lippa, 2009; Schmitt, 2005). More gender equal societies usually have more progressive social roles and ideologies, as well as better distributed resources, particularly towards women, and lower environmental stress. Consequently, biparental care is less necessary than in less egalitarian societies and unpredictable environments (Lippa, 2009; Schmitt, 2005). As women's mating strategy shifts according to environmental demands, in an environment with lower stress women invest more in mating effort, short-term relationships, and consequently, possibly gain in offspring's genetic variation (Schmitt, 2005). Similarly, women engage in more casual sex in societies with a higher proportion of women than men, which applies in particular to women with lower mate value (Millar et al., 2019; Schmitt, 2005). The less numerous sex becomes the limiting factor, being more valuable and obtaining more bargaining power within the local mating market. These advantages allow the less numerous sex to pursue its favoured strategy. In this case, rare males can avoid or minimise investment in one partner while seeking low-investment copulation with multiple partners (Lippa, 2009; Schmitt, 2005).

Mixed results have been reported about association among sociosexuality, mate value, intrasexual competition and/or mental health. Some studies pointed out that higher sociosexual behaviour in women was associated with worry-vulnerability (Townsend & Wasserman, 2011), sexual victimisation (Townsend et al., 2020), more depressive symptoms (Grello et al., 2006) and psychological distress (Fielder & Carey, 2010). Higher sociosexuality also decreases relationship quality (French et al., 2019). However, the association between casual sex and indicators of well-being can also be positive in both sexes (e.g. Schmitt & Jonason, 2019) and can be moderated by several factors. Vrangalova and Ong (2014) found that unrestricted men and women presented higher psychological well-being (higher self-esteem and life satisfaction; lower depression and anxiety) after uncommitted sex, while there was no such association among restricted individuals. Further, individuals with extrinsic motivation to casual sex presented lower well-being after casual hook-ups, while no such association appeared in individuals with intrinsic motivations towards casual sex (Vrangalova, 2015).

Worry/vulnerability and emotional upset do not necessarily result from women's casual sexual encounters, nor do they appear in the usual measures of well-being. Instead, women experience upset when there is a discrepancy between what they want/expect from a partner and what they are getting. Women who accept that hookups entail little or no investment do not necessarily experience upset. If they have regular sexual intercourse with a specific partner, however, they will tend to bond and then desire control over his level of commitment and investment (Townsend, 1987, 1998; Townsend et al., 1995). However, women who engage in multiple casual encounters for years tend to exhibit negative psychological symptoms (Furman & Collibee, 2014).

Previous studies mostly focused on casual sex or sociosexual behaviour. However, sociosexual behaviour can differ from sociosexual desires and attitudes. In particular, the behavioural dimension is an outcome of individual desires, combined with personal and external factors that limit the outcome realisation (Penke & Asendorpf, 2008). Sociosexual attitudes, on the other hand, are evaluations of sexuality, reflecting the sociocultural values of the given population. For example, a person can have a very high sexual desire (fantasies, arousal), a much lower frequency of sociosexual behaviour (e.g. because of unavailability of casual sexual partners) and very restricted attitudes (e.g. because the local religion values sexual chastity). A possible association between well-being and dimensions of sociosexuality other than behaviour (i.e. desire and attitudes) is yet to be tested. For example, Sociosexual Orientation Inventory (SOI) attitudes did not predict female sexual victimisation but SOI behaviour did (Townsend et al., 2015).

Similarly, women with higher intrasexual competition and lower mate value presented several negative psychological symptoms such as disordered eating behaviours (Abed et al., 2012; Workman & Reader, 2014) and higher depression scores (Kirsner et al., 2003). It might thus be expected that higher intrasexual competition also decreases individual well-being. Contrarily, women with higher self-perceived physical attractiveness (i.e. one of the components of women's mate value) reported higher happiness (Gupta et al., 2016; Ko & Suh, 2019). However, mate value is a broader concept than physical attractiveness, and its association with well-being is unknown.

Furthermore, depression increases with age in women between 15 and 55 years. Some authors report that this relationship becomes negative when women are over 55 years (usually after menopause), but there are others that report higher rates of depression in older women, even after menopause (for discussion see, Faravelli et al., 2013). Some authors suggest that the age-dependent variation in depression can be explained through hormonal variation, pointing out that there is an increased risk for depression associated with changes in androgen and estrogen levels (Solomon & Herman, 2009; Oldehinkel & Bouma, 2011). Nevertheless, socio-cultural influences on women's behaviour during aging can play an important role as well (Oldehinkel & Bouma, 2011).

Our aim is to investigate which of the evolutionary reproduction-related variables (i.e., mate value, intrasexual competition, sociosexuality, relationship status and age) would predict women's mental health (i.e., well- and ill-being). We predict that mate value would positively predict well-being, whereas intrasexual competition and sociosexuality would negatively predict well-being. The opposite would be true for depression. The study is mostly exploratory because the above outlined literature did not jointly address the current independent variables in a single model.

Importantly, most previous studies on mating strategies and well-being were conducted in North American and West European populations, i.e. in Western, Educated, Industrialised, Rich and Democratic (WEIRD) societies, thus being far from representative of the human species (Apicella et al., 2020; Henrich et al., 2010). Asian, African and Latin American populations are less frequently studied, despite being populous and highly genetically and culturally diverse (Rad et al., 2018). Here we present data on a less-WEIRD population of Brazilian women, who are among the most anxious and depressive in the world (McPhillips, 2016), thus presenting an important opportunity to study women's well-being.

## 2. Methods

### 2.1. Participants

A total of 1,547 participants took part in the research. In the final analyses, we included 1,173 cisgender predominantly heterosexual women of 18 years or older (mean, *M*_age_ = 31.89; standard deviation, SD_age_ = 11.10). The majority of our sample were from southeast Brazil and self-identified as white (73.8%), *pardo* (or mixed-race, 17.9%), Black (3.6%), Asian (2.9%), Indigenous (0.6%) or belonging to other ethnicities (1.2%). Around one-third of the participants (34.3%) had completed graduate studies, 23.9% were undergraduate students, 21.4% had graduated, 9.9% had finished high school, 9% were graduate students and 1.6% had not completed elementary school. Regarding family income per month, most of the participants (27.4%) earned USD730–1,470 (R$2.811–5.622), 19.5% earned USD 1,470–2,200 (R$5,622–8,433), 14.9% earned above USD3,660 (above R$14,055), 14.4% earned USD 240–730 (R$937–2,811), 11.8% earned USD 2,200–2,930 (R$ 8,433–11,244), 7.9% earned USD2,930–3,660 (R$11,244–14,055) and 3.1% earned less than USD240 (R$937).

### 2.2. Instruments

The study was part of a larger project focused on makeup usage, well-being, and personality in women (Mafra et al., 2019), and only questionnaires relevant for this study are presented below (all instruments are provided in Supplementary Material 1 in Brazilian Portuguese and English versions). For descriptive statistics of the studied variables, see Table 1.

**Table 1.** Descriptive statistics of the studied variables

Variables	*N*	Minimum	Maximum	Mean	SD	Variance
Age	1173	18.00	72.00	31.89	11.10	123.30
Depression	839	8.00	32.00	18.15	5.54	30.72
Happiness	1020	4.00	28.00	18.61	5.46	29.79
Life satisfaction	1076	4.00	28.00	17.38	6.13	37.53
Mate value	909	1.00	7.00	4.98	1.08	1.18
Intrasexual competition	863	1.00	6.83	1.99	0.94	0.89
SOI desire	858	3.00	27.00	8.26	5.24	27.51
SOI behavior	862	3.00	26.00	8.05	5.18	26.85
SOI attitude	859	3.00	27.00	14.44	7.68	58.95
SOI global	855	3.00	76.00	30.80	14.46	209.02

Note: Differences in sample sizes are due to incomplete questionnaires. SOI, Sociosexual Orientation Inventory.

#### 2.2.1. Sociodemographic questionnaire

The sociodemographic questionnaire included questions about age, sex and gender, sexual orientation, ethnicity, social status (family income and education) and relationship status. Relationship status was a dichotomous variable, with participants indicating if they were currently in a stable relationship. The majority of the participants were in a stable relationship (63.7%), while 36.3% of the women were single.

#### 2.2.2. The Center of Epidemiological Studies Depression Scale

The Center of Epidemiological Studies Depression Scale (CESD-8) is a reduced version of the original Center of Epidemiological Studies Depression Scale (Bracke et al., 2008) used to identify populations under risk of depression. This instrument is composed of eight items that measure, on four-point Likert scales, how often in the last week participants felt depressed, lonely, sad, happy or tired, enjoyed life, slept restlessly or felt that they could not continue. We used the version translated (translated/back-translated) into Brazilian Portuguese (Cronbach *α*= 0.845).

#### 2.2.3. The Subjective Happiness Scale

The Subjective Happiness Scale (SHS) is a four-item measure that evaluates happiness on a seven-point responses scale (Lyubomirsky & Lepper, 1999). We used the version translated and validated for Brazilian Portuguese by Damásio et al. (2014). An example of an item is: ‘In general, I consider myself’ with the options ranging from ‘(1) not a very happy person’ to ‘(7) a very happy person’ (Cronbach *α* = 0.826).

#### 2.2.4. Satisfaction with Life Scale

The Satisfaction with Life Scale (SWLS) is a five-item instrument that measures the cognitive domain of life satisfaction on a seven-point response scale (Diener et al., 1985). We used the version translated and validated for Brazilian Portuguese by Giacomoni and Hutz (1997). An example of a item is: ‘In most ways, my life is close to my ideal’ (Cronbach *α* = 0.887).

#### 2.2.5. Self-perceived Mate Value questionnaire

The Self-perceived Mate Value questionnaire includes four items that are answered on a seven-point scale (Edlund & Sagarin, 2014). An example of a question is: ‘Overall, how would you rate your level of desirability as a partner?’ We used the translated (translation/back-translation) version into Brazilian Portuguese (Cronbach *α* = 0.820).

#### 2.2.6. Intrasexual Competition Scale

The Intrasexual Competition Scale includes 12 items that are answered on a seven-point scale; participants report how much the intrasexual competition tendency applies to them (1 = not applicable at all, 7 = very much applicable) (Buunk & Fisher, 2009). An example item is: ‘I wouldn't hire a very attractive woman as a colleague’. We used the translated (translation/back-translation) version into Brazilian Portuguese (Cronbach *α* = 0.882).

#### 2.2.7. Revised Sociosexual Orientation Inventory

The Revised Sociosexual Orientation Inventory (SOI-R) (Penke & Asendorpf, 2008) is a nine-item instrument that measures individuals’ tendency towards engaging in casual sexual variety without emotional investment. Higher scores indicate unrestricted sociosexual orientation. This scale was validated for Brazilian Portuguese by Nascimento et al. (2018); however, we used the translated version (translated/back-translated) into Brazilian Portuguese since this project started before the publication of the validation. The scale is computed as a global sociosexual orientation (Cronbach *α* = 0.828), and further divided into three subscales measuring sociosexual behaviour (Cronbach *α* = 0.785), attitude (Cronbach *α* = 0.783) and desire (Cronbach *α* = 0.728).

### 2.3. Procedure

The anonymous volunteers were recruited through social media (e.g., Facebook, Instagram) and institutional e-mails. The inclusion criteria were to be a Brazilian woman over 18 years old and to have access to a computer/tablet/cell phone with internet connection. First, the participants agreed with the consent term, then they responded to anonymous online questionnaires via Qualtrics (Qualtrics, Provo, UT, USA). Participants took around 45 min to complete the entire survey. The study was approved by the IRB of the Institute of Psychology, University of Sao Paulo (no. 90370517.1.0000.5561). The data were collected during 2018–2019, before the outbreak of the COVID-19 pandemic that disrupted much of normal routine and decreased well-being (e.g., Zacher & Rudolph, 2020).

### 2.4. Data analyses

From the 1,547 initial participants, we included 1,173 cisgender predominantly heterosexual women of 18 years or older (*M*_age_ = 31.89; SD_age_ = 11.10); thus transgender, predominately non-heterosexual (e.g., bisexual or homosexual) women and those younger than 18 years old did not enter into the final analyses. First, using IBM SPSS Statistics for Windows, version 21 (IBM Corp., Armonk, NY, USA), we checked data normality. Most data were not normally distributed, and we thus conducted non-parametric correlations and categorical regressions. We ran non-parametric Kendall correlations because this analysis is more resistant to outliers (Croux & Dehon, 2010). To explore the data and verify correlations among the independent variables that implied the possibility of multicollinearity among independent variables, we correlated well-being measures (i.e., life satisfaction and happiness), ill-being (i.e., depression), age, intrasexual competition, mate value and sociosexuality (i.e., global sociosexuality, and sociosexual desire, behaviour and attitude). The independent variables are only weakly and moderately associated which discounted multicollinearity. This was further supported by the variance inflation factors that were not near the value of 10 and the average variance inflation factor was not greater than 1.

To investigate more directly whether reproduction evolution-relevant variables predicted women's well- or ill-being, we conducted categorical regressions because: (a) this method uses an optimal scaling feature that solves the problem of lack of linearity of the scales; and (b) it calculates an optimal regression equation and the effect of each independent variable (i.e., mate value, intrasexual competition, sociosexuality, relationship status and age) on the dependent variables (i.e., depression, life satisfaction and happiness). For these analyses, we used the three dimensions of sociosexuality instead of the global sociosexuality. Scale variables were treated as numerical. For all the analyses, we used a 95% confidence interval.

## 3. Results

### 3.1. Non-parametric (Kendall's) correlations among measures of well-being, sociosexuality, intrasexual competition, mate value and age

The results showed a moderately negative correlation among depression and well-being measures, and a strongly positive correlation between the two well-being measures (i.e., life satisfaction and happiness). Depression was negatively and weakly associated with mate value and age, and positively and weakly correlated with intrasexual competition, global sociosexuality and sociosexual desire. Happiness and life satisfaction were positively and moderately/weakly associated with mate value and age, and negatively and weakly correlated with intrasexual competition, global sociosexuality and sociosexual desire.

Age was positively and weakly correlated with sociosexual behaviour and negatively and weakly correlated with intrasexual competition. There was no correlation between intrasexual competition and mate value. Intrasexual competition was positively and weakly associated with global sociosexuality, sociosexual desire and sociosexual behaviour. Mate value was positively and weakly associated only with sociosexual behaviour.

For further details, see the whole correlation matrix in Table S1 (Supplementary Material).

### 3.2. Evolutionary predictors of women's well-being (categorical regressions)

To test if well-being would be positively predicted by mate value and negatively predicted by intrasexual competition and sociosexuality, and if depression would be positively predicted by intrasexual competition and sociosexuality and inversely predicted by mate value, we conducted categorical regressions.

The results indicated that depression was negatively predicted by mate value and age, and positively by intrasexual competition and sociosexual desire. In contrast, happiness was positively predicted by mate value, marginally positively predicted by relationship status and negatively by intrasexual competition and sociosexual desire. Life satisfaction was positively predicted by mate value (see Table 2). Thus, women with higher self-perceived mate value and lower intrasexual competition scored higher on well-being measures and lower on depression.

**Table 2.** Summary of the categorical regressions ran for ill- and well-being

	Model resume		ANOVA				Coefficients			
	*R*	*R*^2^	*F*	d.f.	*P*		*B*	d.f.	*F*	*P*
Depression										
	0.465	0.216	11.961	18	≤0.001	Age	**−0**.**151**	**3**	**13**.**662**	**≤0**.**001**
						Mate value	**−0**.**358**	**4**	**54**.**103**	**≤0**.**001**
						Intrasexual competition	**0**.**146**	**3**	**13**.**615**	**≤0**.**001**
						SOI desire	**0**.**149**	**4**	**8**.**926**	**≤0**.**001**
						SOI behaviour	−0.007	1	0.006	0.941
						SOI attitude Relationship status	−0.053 −0.021	1 2	0.680 0.340	0.410 0.712
Happiness										
	0.530	0.281	26.552	12	≤0.001	Age	0.041	1	0.433	0.511
						Mate value	**0**.**470**	**3**	**134**.**244**	**≤0**.**001**
						Intrasexual competition	**−0**.**117**	**2**	**13**.**000**	**≤0**.**001**
						SOI desire	**−0**.**101**	**3**	**4**.**691**	**0**.**009**
						SOI behaviour	−0.071	1	1.023	0.312
						SOI attitude	0.089	1	1.123	0.290
Life satisfaction						Relationship status	0.061	2	2.836	0.059
	0.417	0.174	12.159	14	≤0.001	Age	0.084	2	1.021	0.361
						Mate value	**0**.**368**	**4**	**99**.**912**	**≤0**.**001**
						Intrasexual competition	−0.052	2	1.216	0.297
						SOI desire	−0.087	2	2.128	0.120
						SOI behaviour	−0.081	2	1.859	0.156
						SOI attitude Relationship status	0.105 0.060	1 1	1.124 2.339	0.289 0.127

Note: Statistically significant values are in bold (*P* ≤ 0.05).

### 3.3. Additional analyses

Because of the divergence among previous results on the relationship between age and well-being, we ran additional analysis to estimate better curve fit to our data. Age entered the curve estimation as an independent variable and depression, happiness and life satisfaction as dependent variables.

The cubic model explains depression the best (*R*^2^ = 0.047; *F* (3, 835) = 13.757; *P* ≤ 0.001; constant = 37,771; *b*_1_ = −1,547; *b*_2_ = 0.039; *b*_3_ = 0.000). It indicates that in this study depression decreased from 18 years of age until approximately age 25. Then it remained stable until around 50 years, when it fell again (see Figure S1 in Supplementary Material).

The inverse model fits best the relation between age and happiness (*R*^2^ = 0.012; *F* (1, 1018) = 12.00; *P* = 0.001; constant = 20.519; *b*_1_ = −55.137), suggesting that between 18 and 30 years of age happiness levels grow more sharply than after 30 years (Figure S2). Life satisfaction also increases with time, but rather linearly (*R*^2^ = 0.007; *F* (1, 1074) = 7.548; *P* = 0.006; constant = 15.887; *b*_1_ = 0.047; Figure S3).

## 4. Discussion

In this study, we investigated associations among well-being (i.e., happiness and life satisfaction), ill-being (i.e., depression) and evolutionary reproduction-related factors (i.e, mate value, intrasexual competition, sociosexuality and age) in a large sample of a less-WEIRD sample of Brazilian women. Overall, women who scored higher in mate value and lower in intrasexual competition and sociosexual desire reported higher well-being and lower depression; age affected depression negatively.

Women with higher sociosexual desire (but not attitudes or behaviour) had lower well-being. Sociosexual desire usually shows the biggest sex difference with men outscoring women (del Río et al*.,* 2019). It is related to dissatisfaction with sexual life and is negatively related to relationship commitment (Barrada et al., 2018). Furthermore, women who did not conform to traditional gender roles (i.e. masculine women) reported higher sociosexuality (Bartova et al., 2020).

Short-term mating has multiple costs, such as sexually transmitted infections and lack of parental investments. Hence, it is a viable route toward fitness, albeit a risky one. Although women can benefit from short-term mating in terms of resources or offspring genetic variability, they mostly cannot increase their reproductive success with higher numbers of sex partners as much as men, and one might thus expect their greater selectivity of long-term partners (Buss & Schmitt, 2019). In general, women and men invest more into parenting than mating, indicatig that long-term relationships offer a safer route towards reproductie advantages (Valentova et al., 2020). Thus, sexual desire focused more on a long-term partner might, on average, improve personal satisfaction, whereas desire for uncommitted casual encounters might show the opposite trend. Indeed, although women reported not being concerned about their sexual partner's intentions and did not differ in number of sexual partners from men, they were still concerned about their partner's feelings for them and felt more vulnerable after having uncommitted sex (Hehman & Salmon, 2020). These results suggest that, despite women's willingness to engage in uncommitted sexual relationships, they present psychological mechanisms that made them more sensitive when engaging in a sexual relationship with a partner who did not want to invest more in them (Townsend et al., 2015).

Proximate social factors can also be at play. In particular, a double standard rewarding casual sex in men and discouraging such behaviour in women (Rudman et al., 2017) might lead to lower well-being among women who have, for instance higher sex drive aimed at a variety of sexual partners or unsecure attachment style.

Intrasexual competition had a negative impact on well-being and increased with depression. Consonant with the current study, Gilbert et al. (2009) observed that in depressed individuals the need to avoid inferiority seems to be stronger, particularly when individuals evaluate themselves as belonging to a low social rank. Intrasexual competition in professional and social environments may trigger ill-being, including depression. In general, depression can be a reaction to one's fear of rejection when feeling subordinate (Gilbert et al*.,* 2009; Langner et al*.,* 2012).

Depression seems to be also linked to intrasexual competition for mates. Higher tendency/necessity to compete over partners might be, on the proximate level, accompanied by feelings of insufficiency, which decreases self-esteem and overall well-being. Moreover, whenever intrasexual competition is intense (e.g., in an environment with a higher proportion of women than men), lower-mate-value women are more prone to adopting an unrestricted mating strategy (Millar et al*.,* 2019). For example, Kirsner et al. (2003) reported that participants with higher depression scores evaluated themselves as having lower mate value, and lower-mate-value women tend to accept a higher number of potential romantic partners, including ones with similar mate value (Lee et al., 2008). In contrast, higher-mate-value women tend to be more demanding when choosing their romantic partners (Buss & Shackelford, 2008). Thus, we might assume that lower perceived mate value might increase the necessity to compete over partners, which decreases subjective well-being.

Indeed, women with lower self-reported mate value scored lower on well-being and higher on depression. Mate value is positively associated with self-esteem (Mafra & Lopes, 2014), which, in turn, is also positively correlated with well-being (e.g., Dogan et al*.,* 2013). Indeed, some researchers consider self-esteem to be a measure of psychological well-being (e.g., Vrangalova & Ong, 2014). Moreover, low-mate-value individuals tend to use more cost-inflicting mate retention behaviours while high-mate-value individuals tend to use more benefit-provisioning mate retention behaviours (Miner et al., 2009; Salkicevic et al*.,* 2014). Cost-inflicting behaviours and low mate value are negatively correlated with relationship satisfaction whereas benefit-provisioning mate retention behaviours and high mate value are positively correlated with relationship satisfaction (Salkicevic et al., 2014). Dush and Amato (2005) found positive correlations among life satisfaction, life happiness and relationship happiness, suggesting that the cost-inflicting behaviours used by low-mate-value partners decrease well-being. In fact, Hromatko et al. (2015) found that the higher the mate value of an individual's partner, the higher the relationship satisfaction. Thus, women with higher mate value would attract high-mate-value partners more easily and would use more benefit-provisioning tactics to retain their partners, increasing well-being. These tactics diminish the necessity for intrasexual competition and orientation towards uncommitted sex, reducing ill-being and increasing well-being.

We found that relationship status marginally positively predicted happiness, which is in line with a previous study showing that the higher the relationship commitment is, the higher the subjective well-being level (i.e., married individuals reported the highest well-being scores; Dush & Amato, 2005). Altogether, these findings suggest that a long-term committed sexual strategy may be related to higher well-being and lower ill-being. Indeed, long-term romantic bonds are universal and a prevailing human mating system (Fletcher et al., 2015), and there is some evidence that relationship commitment increases reproductive success in naturally fertile populations (Sorokowski et al., 2017). Further, long-term mating orientation is positively correlated to mate value and slow life history (Strouts et al., 2017) and couples with similar mate value tend to have a more lasting relationship and report higher relationship satisfaction (Salkicevic et al., 2014).

In the same vein, women in our sample who were more concerned about mating (i.e., higher sociosexuality and intrasexual competition) presented lower scores of happiness and higher scores of depression. Accordingly, among individuals whose well-being was related to their meaning of life, kin-care motivation had higher importance than mate-seeking motivation (Ko et al., 2019). In comparison, in participants whose well-being was related to maximising pleasures in life, mate-seeking had the same importance as kin-care. That is, even among individuals who linked their well-being more to pleasures (i.e., high sociosexuality), mate-seeking (i.e., lower sociosexuality) was still not more important than kin-care (Ko et al., 2019). Additionally, individuals who reported high levels of kin-care motivation also reported higher life satisfaction and lower depression whereas the opposite (i.e., low life satisfaction and high depression indexes) was found in participants who presented high mate-seeking motivation (Ko et al., 2019).

Finally, age negatively predicted depression. We found that a non-linear (cubic) curve described the relationship between age and depression. Depression decreased between 18 and approximately 25 years, then remained stable until 50 years, when depression seemed to diminish again (Figure S1, Supplementary Material). These findings corroborated previous studies (Mirowsky & Ross, 1992). Indeed, according to the National Institute of Mental Health (2017), depression seems to be most common among adolescent US women (20%). In Brazil, between 2015 and 2018 the incidence of depression increased by 115% in the age group of 15–29 years, according to a survey by the Ministry of Health (G1, 2019).

## 5. Limitations and future directions

Despite having a large sample, we used a cross-sectional study design, which limits causal explanation. Future research could use experimental design, longitudinal study design or more specific questions about the circumstances that drive well- and ill-being. We did not have a sample large enough to analyse women older than 50 years to better investigate the relationship between age and well-being. Also, our sample relies on cisgender predominantly heterosexual women mainly from the state of Sao Paulo, thus restricting our results to a non-representative portion of the Brazilian population. Minorities are still targets of discrimination and show lower well-being (Perales, 2016), poor self-esteem and high ill-being (Witcomb et al., 2019). Studies including non-heterosexual women and transgender women would improve our understanding of feminine reproduction-related aspects (Valentova & Varella, 2016) and provide more information to improve well-being of this part of the population. Also, our sample was composed of mostly self-declared white participants. Future studies would benefit from recruiting a more ethnically diverse sample or focusing on non-white populations that might be of higher risk of decreased well-being.

## 6. Conclusion

We showed that women's mate value and age are positively associated with well-being and negatively associated with ill-being, whereas intrasexual competition and sociosexual desire exhibited the opposite trend. Our results suggest that strategies which can increase women's reproductive success under some circumstances do not necessarily lead to feelings of well-being (for example, when women are competing with high-mate-value rivals). Thus, in women, subjective well-being might trace fitness enhancement mainly through the long-term relationship strategy rather than through mating efforts.

## Supplementary material

For supplementary material accompanying this paper visit https://doi.org/10.1017/ehs.2021.18. click here to view supplementary material

## Research transparency and reproducibility

The data is available at: DOI 10.17605/OSF.IO/PDSXZ
