# Supplementary material for: Mate value, intrasexual competition and sociosexual desire drive Brazilian women's well-being
Source: Evol Hum Sci. 2021 Mar 10;3:e25. doi: 10.1017/ehs.2021.18 (PMC10427315; doi:10.1017/ehs.2021.18)
Supplement: Supplementary file 1 [file S2513843X21000189sup.zip › S2513843X21000189sup001.docx]

Supplementary material 1

**2.2.1. Questionário sociodemográfico**

1. Qual o seu nível de escolaridade? (Por favor, circule apenas o grau mais elevado )

Ensino fundamental incompleto

Ensino fundamental completo

Escola técnica completa

Ensino médio completo

Cursando graduação atualmente

Graduação completa

Cursando mestrado atualmente

Mestrado completo

Cursando doutorado atualmente

Doutorado completo

2. Somando a sua renda com a das pessoas que moram com você, quanto é, aproximadamente, a renda familiar mensal?

Nenhuma renda

1 salário mínimo (R$ 937)

1-3 salários mínimos (R$ 937 a 2.811)

3-6 salários mínimos (R$ 2.811 a 5.622)

6-9 salários mínimos (R$ 5.622 a 8.433)

9-12 salários mínimos (R$ 8.433 a 11.244)

12-15 salários mínimos (R$ 11.244 a 14.055)

Mais de 15 salários mínimos (acima de R$ 14.055)

3. Qual a sua raça?

Negra

Parda

Branca

Oriental

Indígena

Outra (por favor, especifique)

4. Qual o seu sexo de nascença?

Masculino

Feminino

Outro

5. Com qual gênero você se identifica?

Homem

Mulher

Outro

6. Qual das seguintes opções melhor descreve seu atual status de relacionamento ?

Não estou namorando atualmente

Ficando com apenas uma pessoa (sem namorar)

Ficando com mais de uma pessoa

Namorando mais de uma pessoa

Namorando uma pessoa

Morando com meu/minha parceiro/a

Casado/a

Casado/a de novo

7. Há quanto tempo você está com seu/sua parceiro/a principal?

Menos de 3 meses

De 3 a 6 meses

De 6 meses a 1 ano

De 1 a 5 anos

De 5 a 10 anos

De 10 a 15 anos

De 15 a 20 anos

De 20 a 25 anos

De 25 a 30 anos

Mais de 30 anos

8. Qual é o gênero do seu/sua parceiro/a principal? (Selecione todas as opções que se aplicam)

Homem

Mulher

Outro

9. Em uma escala de 0 a 10, o quanto você se sente sexualmente atraído(a) por homens?

Nenhuma atração 0 123456789 10 Muita atração

10. Em uma escala de 0 a 10, o quanto você se sente sexualmente atraído(a) por mulheres?

Nenhuma atração 0 123456789 10 Muita atração

1. Quanto dinheiro você gasta em cosméticos por mês?

Less than $10 (± R$ 31,50)

$11-25 (± R$ 34,69 a 78,83)

$26-40 (± R$ 81,99 a 126,14)

$41-60 (± R$ 129,29 a 189,20)

$61-80 (± R$ 192,36 a 252,27)

$81-100 (± R$ 255,43 a 315,34)

More than $100 (+ R$ 315,34)

2. Quantas vezes você compra cosméticos por mês?

0

1

2-3

4-5

More than 5

3. Quantas vezes você compra roupas e acessórios por mês?

0

1

2-3

4-5

More than 5

4. Quanto dinheiro você gasta em roupas e acessórios por mês?

Less than $20 (± R$ 63,07)

$21-$50 (± R$ 66,22 a 157,67)

$51-$100 (± R$ 160,82 a 315,34)

$101-$150 (± R$ 318,49 a 473,01)

$151-$200 (± R$ 476,16 a 630,68)

More than $200 (+ R$ 630,68)

5. Quando tempo você gasta aplicando cosméticos por dia?

Menos que 5 minutos

5-10 minutos

10-20 minutos

20-30 minutos

Mais que 30 minutos

6. Quando tempo você gasta aplicando cosméticos em ocasiões especiais (festas, sair, encontros, etc.)?

Menos que 5 minutos

5-10 minutos

10-20 minutos

20-30 minutos

Mais que 30 minutos

**2.2.1. Sociodemographic questionnaire**

1. What is your level of education? (Please circle only the highest grade)

Incomplete Elementary school

Complete Elementary School

Complete Technical school

Complete High School

Currently attending College

Complete College

Currently attending for a master's

Complete Master‘s Degree

Currently attending for a Ph.D.

Complete Ph.D.

2. Adding your income to that of the people who live with you, how much is the monthly family income approximately?

No income

1 minimum wage (R$ 937)

1-3 minimum wages (R$ 937 to 2.811)

3-6 minimum wages (R$ 2,811 to 5,622)

6-9 minimum wages (R$ 5,622 to 8,433)

9-12 minimum wages (R$ 8,433 to 11,244)

12-15 minimum wages (R$ 11,244 to 14,055)

More than 15 minimum wages (above R$ 14,055)

3. What is your ethnicity?

Black

Parda (mixed ethnicity)

White

Oriental

Indigenous

Other (please specify)

4. What is your gender at birth?

Male

Female

Other

5. With which gender do you identify?

Man

Woman

Other

6. Which of the following best describes your current relationship status ?

I am not currently dating

Hanging with only one person (not dating)

Hanging with more than one person (not dating)

Dating more than one person

Dating one person

Living with my partner

Married

Remarried

7. How long have you been with your main partner?

Less than 3 months

From 3 to 6 months

From 6 months to 1 year

From 1 to 5 years

From 5 to 10 years

From 10 to 15 years

From 15 to 20 years

From 20 to 25 years

From 25 to 30 years

More than 30 years

8. What is the gender of your main partner?

Man

Woman

Other

9. On a scale of 0 to 10, how much are you sexually attracted to men?

No attraction 0 1 2 3 4 5 6 7 8 9 10 Highly attracted

10. On a scale of 0 to 10, how much are you sexually attracted to women?

No attraction 0 1 2 3 4 5 6 7 8 9 10 Highly attracted

11. How much money do you spend on cosmetics per month?

Less than $10 (± $ 31.50)

$11-25 (± R$ 34.69 to 78.83)

$26-40 (± R$ 81.99 to 126.14)

$41-60 (± R$ 129.29 to 189.20)

$61-80 (± R$ 192.36 to 252.27)

$81-100 (± R$ 255.43 to 315.34)

More than $100 (+ R$ 315.34)

12. How many times do you buy cosmetics per month?

0

1

2-3

4-5

More than 5

13. How often do you buy clothes and accessories per month?

0

1

2-3

4-5

More than 5

14. How much money do you spend on clothes and accessories per month?

Less than $20 (± R$ 63.07)

$21-$50 (± R$ 66.22 to 157.67)

$51-$100 (± R$ 160.82 to 315.34)

$101-$150 (± R$ 318.49 to 473.01)

$151-$200 (± R$ 476.16 to 630.68)

More than $200 (+ R$ 630.68)

15. How long do you spend applying cosmetics per day?

Less than 5 minutes

5-10 minutes

10-20 minutes

20-30 minutes

More than 30 minutes

16. How long do you spend applying cosmetics on special occasions (parties, going out, dates, etc)?

Less than 5 minutes

5-10 minutes

10-20 minutes

20-30 minutes

More than 30 minutes

**2.2.2. Escala de Depressão do Centro de Estudos Epidemiológicos 8-itens**

| Quanto tempo durante a semana passada... | | | | |
| --- | --- | --- | --- | --- |
|  | 0  Quase nenhuma vez | 1 | 2 | 3  Todo o tempo |
| Você se sentiu deprimido/a? |  |  |  |  |
| Você sentiu que tudo o que você fazia era um esforço? |  |  |  |  |
| Seu sono foi reparador? |  |  |  |  |
| Você se sentiu feliz? |  |  |  |  |
| Você se sentiu solitário/a? |  |  |  |  |
| Você aproveitou a vida? |  |  |  |  |
| Você se sentiu triste? |  |  |  |  |
| Você não conseguiu continuar? |  |  |  |  |

**2.2.2. The Center for epidemiological Studies Depression Scale 8-item (CESD-8) (Bracke, Levecque, & Van de Velde, 2008)**

| How much of the time during the past week... | | | | |
| --- | --- | --- | --- | --- |
|  | 0  Almost none of the time | 1 | 2 | 3  All the time |
| You felt depressed? |  |  |  |  |
| You felt everything you did was an effort? |  |  |  |  |
| Your sleep was restless? |  |  |  |  |
| You were happy? |  |  |  |  |
| You felt lonely? |  |  |  |  |
| You enjouyed life? |  |  |  |  |
| You felt sad? |  |  |  |  |
| You could not get going? |  |  |  |  |

**2.2.3. Escala de Felicidade Subjetiva (Damásio, Zanon & Koller, 2014)**

1. Em geral, eu me considero:

| 1  Uma pessoa não muito feliz | 2 | 3 | 4 | 5 | 6 | 7  Uma pessoa muito feliz |
| --- | --- | --- | --- | --- | --- | --- |

2. Comparado à maioria dos meus colegas/amigos, eu me considero:

| 1  Menos feliz | 2 | 3 | 4 | 5 | 6 | 7  Mais feliz |
| --- | --- | --- | --- | --- | --- | --- |

3. Algumas pessoas, de maneira geral, são muito felizes. Elas aproveitam a vida independentemente do que esteja acontecendo, conseguindo o máximo de cada situação. Em

que medida essa caracterização descreve você?

| 1  Nem um pouco | 2 | 3 | 4 | 5 | 6 | 7  Muito |
| --- | --- | --- | --- | --- | --- | --- |

4. Algumas pessoas, de maneira geral, não são muito felizes. Embora não estejam deprimidas,

elas nunca parecem tão felizes quanto poderiam ser. Em que medida essa caracterização descreve você?

| 1  Nem um pouco | 2 | 3 | 4 | 5 | 6 | 7  Muito |
| --- | --- | --- | --- | --- | --- | --- |

**2.2.3. Subjective Happiness Scale (Lyubomirsky & Lepper, 1999)**

In general, I consider myself:

| 1  A very unhappy person | 2 | 3 | 4 | 5 | 6 | 7  A very happy person |
| --- | --- | --- | --- | --- | --- | --- |

2. Compared to most of my colleagues/friends, I consider myself:

| 1  Less happy | 2 | 3 | 4 | 5 | 6 | 7  Happier |
| --- | --- | --- | --- | --- | --- | --- |

3. Some people, in general, are very happy. They enjoy life regardless of what is happening, getting the most out of each situation. To what extent does this characterization describe you?

| 1  Not at all | 2 | 3 | 4 | 5 | 6 | 7  A lot |
| --- | --- | --- | --- | --- | --- | --- |

4. Some people, in general, are not very happy. Although they are not depressed, they never seem as happy as they could be. To what extent does this characterization describe you?

| 1  Not at all | 2 | 3 | 4 | 5 | 6 | 7  A lot |
| --- | --- | --- | --- | --- | --- | --- |

**2.2.4. Escala de Satisfação com a Vida (Giacomoni & Hutz, 1997)**

Na maioria dos aspectos, minha vida está mais próxima do ideal

| 1  Discordo plenamente | 2  Discordo | 3  Discordo um pouco | 4  Não concordo nem discordo | 5  Concordo um pouco | 6  Concordo | 7  Concordo plenamente |
| --- | --- | --- | --- | --- | --- | --- |

As condições da minha vida são excelentes

| 1  Discordo plenamente | 2  Discordo | 3  Discordo um pouco | 4  Não concordo nem discordo | 5  Concordo um pouco | 6  Concordo | 7  Concordo plenamente |
| --- | --- | --- | --- | --- | --- | --- |

Estou satisfeito/a com minha vida

| 1  Discordo plenamente | 2  Discordo | 3  Discordo um pouco | 4  Não concordo nem discordo | 5  Concordo um pouco | 6  Concordo | 7  Concordo plenamente |
| --- | --- | --- | --- | --- | --- | --- |

Até hoje, consegui as as coisas mais importantes que desejo na vida

| 1  Discordo plenamente | 2  Discordo | 3  Discordo um pouco | 4  Não concordo nem discordo | 5  Concordo um pouco | 6  Concordo | 7  Concordo plenamente |
| --- | --- | --- | --- | --- | --- | --- |

Se pudesse viver minha vida outra vez, não mudaria nada

| 1  Discordo plenamente | 2  Discordo | 3  Discordo um pouco | 4  Não concordo nem discordo | 5  Concordo um pouco | 6  Concordo | 7  Concordo plenamente |
| --- | --- | --- | --- | --- | --- | --- |

**2.2.4. The Satisfaction With Life Scale (Diener, Emmons, Larsen, & Griffin, 1985)**

In most ways my life is close to my ideal

| 1  Strongly disagree | 2  Disagree | 3  Slightly disagree | 4  Neither agree nor disagree | 5  Slightly agree | 6  Agree | 7  Strongly agree |
| --- | --- | --- | --- | --- | --- | --- |

The conditions of my life are excellent

| 1  Strongly disagree | 2  Disagree | 3  Slightly disagree | 4  Neither agree nor disagree | 5  Slightly agree | 6  Agree | 7  Strongly agree |
| --- | --- | --- | --- | --- | --- | --- |

I am satisfied with my life

| 1  Strongly disagree | 2  Disagree | 3  Slightly disagree | 4  Neither agree nor disagree | 5  Slightly agree | 6  Agree | 7  Strongly agree |
| --- | --- | --- | --- | --- | --- | --- |

So far I have gotten the important things I want in life

| 1  Strongly disagree | 2  Disagree | 3  Slightly disagree | 4  Neither agree nor disagree | 5  Slightly agree | 6  Agree | 7  Strongly agree |
| --- | --- | --- | --- | --- | --- | --- |

If I could live my life over, I would change almost nothing

| 1  Strongly disagree | 2  Disagree | 3  Slightly disagree | 4  Neither agree nor disagree | 5  Slightly agree | 6  Agree | 7  Strongly agree |
| --- | --- | --- | --- | --- | --- | --- |

**2.2.5. The Mate Value Scale**

No geral, quão desejável você se vê como parceiro/a?

| 1  Extremamente indesejável | 2 | 3 | 4 | 5 | 6 | 7  Extremamente desejável |
| --- | --- | --- | --- | --- | --- | --- |

No geral, quão desejável outras pessoas te veem como parceiro/a?

| 1  Extremamente indesejável | 2 | 3 | 4 | 5 | 6 | 7  Extremamente desejável |
| --- | --- | --- | --- | --- | --- | --- |

 No geral, quão desejável como parceiro/a você se acha em comparação com outras pessoas?

| 1  Muito abaixo da média | 2  Abaixo da média | 3  Um pouco abaixo da média | 4  Na média | 5  Um pouco acima da média | 6  Acima da média | 7  Muito acima da média |
| --- | --- | --- | --- | --- | --- | --- |

No geral, quão “bom partido” você é?

| 1  Muito ruim | 2  Ruim | 3  Um pouco ruim | 4  Na média | 5  Um pouco bom | 6  Bom | 7  Muito bom |
| --- | --- | --- | --- | --- | --- | --- |

# 2.2.5. The Mate Value Scale (Edlund & Sagarin, 2014)

Overall, how would you rate your level of desirability as a partner on the following scale?

| 1  Extremely undesirable | 2 | 3 | 4 | 5 | 6 | 7  Extremely desirable |
| --- | --- | --- | --- | --- | --- | --- |

Overall, how would members of the opposite sex rate your level of desirability as a partner on the following scale?

| 1  Extremely undesirable | 2 | 3 | 4 | 5 | 6 | 7  Extremely desirable |
| --- | --- | --- | --- | --- | --- | --- |

Overall, how do you believe you compare to other people in desirability as a partner on the following scale?

| 1  Very much lower than average | 2  Lower than  average | 3  Slightly  lower than  average | 4  Average | 5  Slightly  higher than  average | 6  Higher than  average | 7  Very much  higher than  average |
| --- | --- | --- | --- | --- | --- | --- |

Overall, how good of a catch are you?

| 1  Very bad catch | 2  Bad catch somewhat | 3  Bad of a  catch | 4  Average  catch | 5  Somewhat  good of a  catch | 6  Good catch | 7  Very good  catch |
| --- | --- | --- | --- | --- | --- | --- |

**2.2.6. Escala de Competição Intrassexual**

|  | 1  Nem um pouco aplicável | 2 | 3 | 4 | 5 | 6 | 7.  Completamente aplicável |
| --- | --- | --- | --- | --- | --- | --- | --- |
| Eu não suporto quando encontro uma mulher que é mais atraente do que eu |  |  |  |  |  |  |  |
| Quando eu saio, eu não suporto quando os homens prestam mais atenção em uma amiga minha do que em mim. |  |  |  |  |  |  |  |
| Eu costumo procurar características negativas em mulheres atraentes. |  |  |  |  |  |  |  |
| Quando eu estou em uma festa, eu gosto quando os homens prestam mais atenção em mim do que em outras mulheres. |  |  |  |  |  |  |  |
| Eu não contrataria uma mulher muito atraente para ser minha colega de trabalho |  |  |  |  |  |  |  |
| Eu simplesmente não gosto de mulheres muito ambiciosas |  |  |  |  |  |  |  |
| Eu costumo procurar características negativas em mulheres que são bem sucedidas |  |  |  |  |  |  |  |
| Eu não contrataria uma mulher altamente competente para ser minha colega de trabalho |  |  |  |  |  |  |  |
| Eu gosto de ser mais divertida e mais perspicaz do que as outras mulheres |  |  |  |  |  |  |  |
| Eu quero ser apenas um pouco melhor do que as outras mulheres |  |  |  |  |  |  |  |
| Eu sempre quero derrotar as outras mulheres |  |  |  |  |  |  |  |
| Eu não gosto de ver outras mulheres com uma casa melhor ou um carro melhor do que os meus |  |  |  |  |  |  |  |

**2.2.6. Intrasexual Competition Scale (Buunk & Fisher, 2009)**

|  | 1  Nem um pouco aplicável | 2 | 3 | 4 | 5 | 6 | 7.  Completamente aplicável |
| --- | --- | --- | --- | --- | --- | --- | --- |
| I can’t stand it when I meet another woman who is more attractive than I am |  |  |  |  |  |  |  |
| When I go out, I can’t stand it when men pay more attention to a same-sex friend of mine than to me |  |  |  |  |  |  |  |
| I tend to look for negative characteristics in attractive women. |  |  |  |  |  |  |  |
| When I’m at a party, I enjoy it when men pay more attention to me than other women |  |  |  |  |  |  |  |
| I wouldn’t hire a very attractive woman as a colleague |  |  |  |  |  |  |  |
| I just don’t like very ambitious women |  |  |  |  |  |  |  |
| I tend to look for negative characteristics in women who are very successful |  |  |  |  |  |  |  |
| I wouldn’t hire a highly competent woman as a colleague |  |  |  |  |  |  |  |
| I like to be funnier and more quick witted than other women |  |  |  |  |  |  |  |
| I want to be just a little better than other women |  |  |  |  |  |  |  |
| I always want to beat other women |  |  |  |  |  |  |  |
| I don’t like seeing other women with a nicer house or a nicer car than mine |  |  |  |  |  |  |  |

**2.2.7. Inventário de Orientação sociossexual Revisto (SOI-R) (Nasciment****o, Hane****l, Monteir****o, Gouveia****, & Little, 2018)**

1. No último ano (últimos 12 meses), com quantas pessoas diferentes você fez sexo?

0 1 2 3 4 5-6 7-9 10-19 20 ou mais

2. Até onde você lembra, em sua vida, com quantas pessoas você já fez sexo apenas uma vez?

0 1 2 3 4 5-6 7-9 10-19 20 ou mais

3. Pensando na sua vida até agora, com quantas pessoas você já fez sexo sem estar interessado/a em um relacionamento duradouro e com compromisso?

0 1 2 3 4 5-6 7-9 10-19 20 ou mais

4. Tudo bem fazer sexo sem amor.

Discordo totalmente 1 2 3 4 5 6 7 8 9 Concordo totalmente

5. Consigo me imaginar confortável e curtindo fazer sexo casual com parceiros diferentes.

Discordo totalmente 1 2 3 4 5 6 7 8 9 Concordo totalmente

6. Eu não quero fazer sexo com um pessoa até que eu tenha certeza de que npos teremos um relacionamento duradouro e sério.

Discordo totalmente 1 2 3 4 5 6 7 8 9 Concordo totalmente

7. Com que frequência você tem fantasias sobre fazer sexo com alguém com quem você não tem relacionamento romântico compromissado?

1 – nunca

2 – muito raramente

3 – uma vez a cada dois ou três meses

4 – uma vez por mês

5 – uma vez a cada duas semanas

6 – uma vez por semana

7 – várias vezes por semana

8 – quase todos os dias

9 – pelo menos uma vez por dia

8. Com que frequência você fica excitado/a sexualmente quando está em contato com alguém com quem você não tem relacionamento romântico compromissado?

1 – nunca

2 – muito raramente

3 – uma vez a cada dois ou três meses

4 – uma vez por mês

5 – uma vez a cada duas semanas

6 – uma vez por semana

7 – várias vezes por semana

8 – quase todos os dias

9 – pelo menos uma vez por dia

9. No seu dia-a-doa, com que frequência você espontaneamente imagina-se fazendo sexo com alguém que acabou de conhecer?

1 – nunca

2 – muito raramente

3 – uma vez a cada dois ou três meses

4 – uma vez por mês

5 – uma vez a cada duas semanas

6 – uma vez por semana

7 – várias vezes por semana

8 – quase todos os dias

9 – pelo menos uma vez por dia

**2.2.7. Revised Sociosexual Orientation Inventory (SOI-R) (Penke & Asendorpf, 2008)**

1. With how many different partners have you had sex within the past 12 months?

0 1 2 3 4 5-6 7-9 10-19 20 or more

2. With how many different partners have you had sexual intercourse on one and only one occasion?

0 1 2 3 4 5-6 7-9 10-19 20 or more

3. With how many different partners have you had sexual intercourse without having an interest in a long-term committed relationship with this person?

0 1 2 3 4 5-6 7-9 10-19 20 or more

4. Sex without love is OK.

Strongly disagree 1 2 3 4 5 6 7 8 9 Strongly agree

5. I can imagine myself being comfortable and enjoying "casual" sex with different partners.

Strongly disagree 1 2 3 4 5 6 7 8 9 Strongly agree

6. I do not want to have sex with a person until I am sure that we will have a long-term, serious relationship.

Strongly disagree 1 2 3 4 5 6 7 8 9 Strongly agree

7. How often do you have fantasies about having sex with someone you are not in a committed romantic relationship with?

1 – never

2 – very seldom

3 – about once every two or three months

4 – about once a month

5 – about once every two weeks

6 – about once a week

7 – several times per week

8 – nearly every day

9 – at least once a day

8. How often do you experience sexual arousal when you are in contact with someone you are not in a committed romantic relationship with?

1 – never

2 – very seldom

3 – about once every two or three months

4 – about once a month

5 – about once every two weeks

6 – about once a week

7 – several times per week

8 – nearly every day

9 – at least once a day

9. In everyday life, how often do you have spontaneous fantasies about having sex with someone you have just met?

1 – never

2 – very seldom

3 – about once every two or three months

4 – about once a month

5 – about once every two weeks

6 – about once a week

7 – several times per week

8 – nearly every day

9 – at least once a day
