# Supplementary material for: Mate value, intrasexual competition and sociosexual desire drive Brazilian women's well-being
Source: Evol Hum Sci. 2021 Mar 10;3:e25. doi: 10.1017/ehs.2021.18 (PMC10427315; doi:10.1017/ehs.2021.18)
Supplement: Supplementary file 1 [file S2513843X21000189sup.zip › S2513843X21000189sup002.docx]

Table S1: Kendall’s correlations between ill-being, well-being, sociosexuality, mate value, and intrasexual competition

|  |  | SOI desire | SOI behavior | SOI attitude | SOI total | Depression | Happiness | Life satisfaction | Intrasexual competition | Mate value |
| --- | --- | --- | --- | --- | --- | --- | --- | --- | --- | --- |
| Age | τ | -.044 | **.126** | .006 | .025 | **-.128** | **.077** | **.054** | **-.054** | .029 |
|  | p | .071 | **≤.001** | .794 | .282 | **≤.001** | **≤.001** | **.010** | **.027** | .214 |
| SOI desire | τ | - | **.285** | **.359** | **.555** | **.199** | **-.114** | **-.072** | **.167** | .015 |
|  | p | - | **≤.001** | **≤.001** | **≤.001** | **≤.001** | **≤.001** | **.003** | **≤.001** | .534 |
| SOI behavior | τ |  | - | **.380** | **.547** | .005 | -.029 | -.030 | **.060** | **.079** |
|  | p |  | - | **≤.001** | **≤.001** | .852 | .243 | .213 | **.015** | **.001** |
| SOI attitude | τ |  |  | - | **.742** | .021 | -.039 | -.033 | .026 | .027 |
|  | p |  |  | - | **≤.001** | .389 | .106 | .165 | .291 | .270 |
| Global SOI | τ |  |  |  | - | **.053** | **-.069** | **-.053** | **.093** | .039 |
|  | p |  |  |  | - | **.028** | **.003** | **.025** | **≤.001** | .106 |
| Depression | τ |  |  |  |  | - | **-.466** | **-.367** | **.135** | **-.243** |
|  | p |  |  |  |  | - | **≤.001** | **≤.001** | **≤.001** | **≤.001** |
| Happiness | τ |  |  |  |  |  | - | **.425** | **-.123** | **.306** |
|  | p |  |  |  |  |  | - | **≤.001** | **≤.001** | **≤.001** |
| Life satisfaction | τ |  |  |  |  |  |  | - | **-.050** | **.255** |
|  | p |  |  |  |  |  |  | - | **.037** | **≤.001** |
| Intrasexual competition | τ |  |  |  |  |  |  |  | - | -.046 |
|  | p |  |  |  |  |  |  |  | - | .055 |

Note: statistically significant values in bold (*p* ≤ .05)


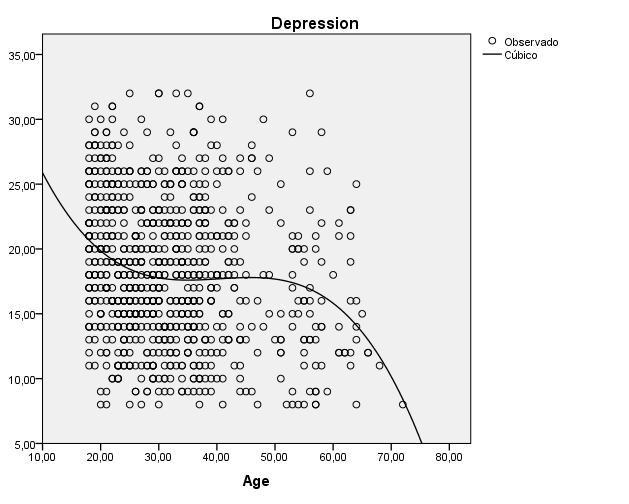


Figure S1: Graphic of the best curve estimation between age and depression


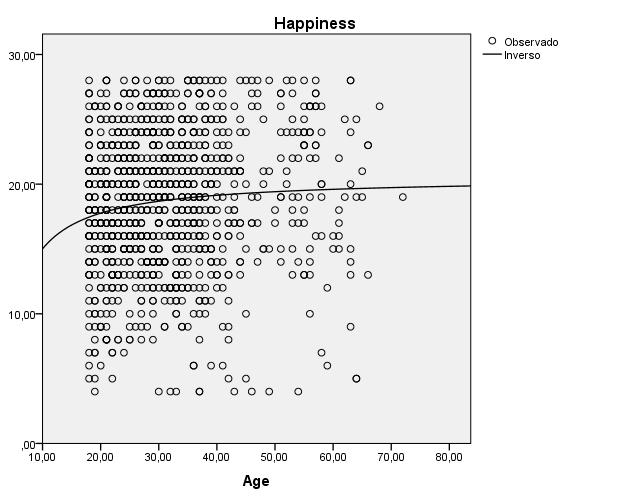


Figure S2: Graphic of the best curve estimation between age and happiness


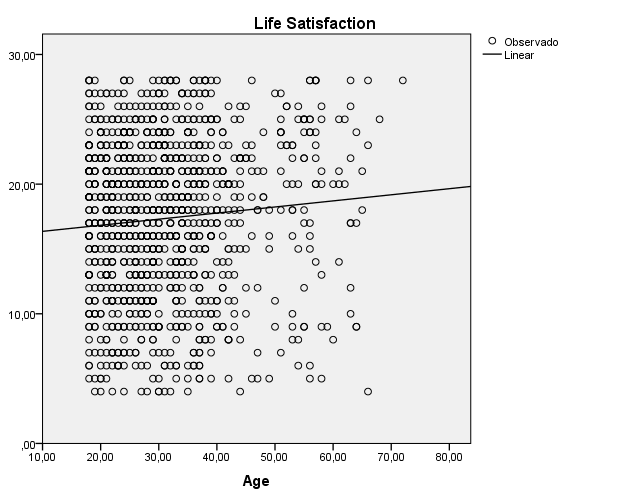


Figure S3: Graphic of the best curve estimation between age and life satisfaction
